# Type and intensity of physical activity and their sex‐specific relation with classroom behaviour in preadolescent children

**DOI:** 10.1111/bjep.70123

**Published:** 2026-08-24

**Authors:** Melanie Berger, Toru Ishihara, Keita Kamijo, Markus Gerber, Sebastian Ludyga

**Affiliations:** ^1^ Department of Sports, Exercise and Health University of Basel Basel Switzerland; ^2^ Graduate School of Human Development and Environment Kobe University Kobe Japan; ^3^ Faculty of Liberal Arts and Sciences Chukyo University Nagoya Japan

**Keywords:** classroom engagement, cognition, gender, physical exercise, sports

## Abstract

**Background:**

On‐task behaviour in the classroom, which partly relies on inhibitory control, contributes to academic achievement. Physical activity has much potential to facilitate on‐task behaviour due to its positive association with inhibitory control. However, it remains unclear how the association between physical activity and on‐task behaviour is influenced by its type and intensity as well as the sex of participants.

**Aim:**

To investigate sex‐specific associations of type and intensity of physical activity engagement and on‐task behaviour as well as its mediation by inhibitory control.

**Sample:**

Cross‐sectional study including 142 participants (47.2% boys) with a mean age of 11.6 years recruited from local schools.

**Methods:**

Classroom behaviour was video‐recorded, with a 6‐min segment coded for on‐task behaviour. Using a 7‐day recall protocol, participants reported type and intensity of physical activity. Additionally, inhibitory control was assessed using a computerized Flanker task.

**Results:**

While path analyses revealed no associations between physical activity intensity, inhibitory control and on‐task behaviour, there was a sex‐specific association with the type of physical activity. More frequent on‐task behaviour was associated with greater participation in open‐skill sports in boys (*β* = .27, *p* < .05) and more engagement in closed‐skill sports in girls (*β* = .25, *p* = .05). However, this association was not mediated by inhibitory control.

**Conclusions:**

The type rather than intensity of physical activity seems to be associated with favourable classroom behaviour. Sex‐specific relations between open‐ and closed‐skill sports and on‐task behaviour highlight the need for interventions tailored specifically to girls and boys.

## INTRODUCTION

### Classroom behaviour and inhibitory control

On‐task behaviour in the classroom is characterized by maintaining attention to the teacher, engaging with the assigned task and adhering to established classroom rules (Brusseau & Burns, 2018; Mahar et al., 2006). Evidence from longitudinal studies suggests that this behaviour has a stronger predictive power for academic and occupational success than familial background, broad personality traits or intelligence (Spengler et al., 2018), highlighting the importance of understanding factors that may promote on‐task behaviour in the classroom. On‐task behaviour involves attending to relevant information while suppressing competing distractions, for example, conversations by fellow students, to sustain a goal‐directed focus. This ability reflects inhibitory control, conceptualized as a common component of executive function, which serves to resist distractions on attentional, emotional and behavioural levels and suppress a prepotent motor response (Miyake & Friedman, 2012). Evidence suggests a link between on‐task behaviour and inhibitory control as children with lower performance in this cognitive domain are more likely to speak out of turn during class (Ludyga et al., 2022) or to engage with non‐relevant material (Moffett & Morrison, 2020). Consequently, improvements in inhibitory control may promise transfer effects to on‐task behaviour.

### Physical activity and on‐task behaviour

Physical activity, which is defined as ‘any bodily movement produced by skeletal muscle that results in energy expenditure’ (Caspersen et al., 1985, p. 126), has the potential to influence on‐task behaviour directly or indirectly and at the same time, it can be implemented in the school setting. A review of the evidence on the direct association between physical activity and on‐task behaviour reveals that the specific modalities of physical activity underpinning this relation are not yet sufficiently characterized, thus limiting their usefulness for the design of targeted interventions. In this regard, Mahar et al. (2006) reported acute and chronic improvements in on‐task behaviour following a 12‐week classroom‐based physical activity program. However, physical activity was assessed only through in‐school step counts as a simple proxy, providing no information on activity intensity or type, and the parallel analysis of physical activity and classroom behaviour precluded conclusions regarding causal mechanisms. Similarly, Carlson et al. (2015) found that classroom activity breaks increased school‐day moderate‐to‐vigorous physical activity (MVPA) and improved teacher‐reported behaviour, although the activities implemented by the teachers were not monitored. Brusseau and Burns (2018) further showed that higher naturally occurring school‐day MVPA was associated with better classroom behaviour, but without characterizing the types of activities performed or accounting for physical activity outside school hours. Notably, these studies predominantly focused on younger children (around 8 years of age), who typically exhibit higher levels of MVPA than preadolescents, given the documented decline in MVPA across school years (Sasayama et al., 2025). This age‐related pattern suggests that the potential to influence on‐task behaviour through increases in MVPA may be particularly relevant during the transition to adolescence, highlighting the need for research specifically focusing on this developmental period.

### Physical activity intensity and type as distinct predictors of inhibitory control

Ludyga et al. (2022) demonstrated that daily MVPA was positively associated with classroom behaviour, with inhibitory control partially mediating this relationship. Thus, higher MVPA was associated with greater on‐task behaviour via enhanced inhibitory control. Building on this rationale, adopting a differentiated perspective on physical activity appears warranted, as both physical activity intensity and type exert distinct effects on inhibitory control, and thus potentially on on‐task behaviour.

Compared to light‐ or moderate‐intensity physical activity, vigorous‐intensity physical activity demonstrates a stronger association with both cardiorespiratory (Ávila‐García et al., 2020; Denton et al., 2013; García‐Hermoso et al., 2021) and muscular fitness (Martínez‐Gómez et al., 2011; Smith et al., 2019). Children with higher levels of cardiorespiratory fitness exhibit superior inhibitory control relative to lower‐fit children (Kao et al., 2017; Pontifex et al., 2011). Cross‐sectional neuroelectric evidence, derived from electroencephalogram event‐related potentials, suggests that lower fitness levels are associated with impairments in cognitive control. These impairments include increased response conflict, indicated by increased N2 amplitude, reduced attentional resource allocation, reflected by smaller P3 amplitude and slower cognitive processing, evidenced by prolonged N2 and P3 latencies in lower‐fit children (Pontifex et al., 2011). Further cross‐sectional evidence for the cognitive benefits of higher cardiorespiratory fitness levels is provided by findings of enhanced task‐induced functional connectivity within the cingulo‐opercular network serving cognitive control, with higher‐fit children showing greater connectivity along specific pathways within this network. The cingulo‐opercular network comprises anterior regions of the cingulate cortex, prefrontal cortex and insula and is characterized by sustained activity during goal‐directed tasks, likely supporting the regulation of goal‐directed behaviour (Ligeza et al., 2024). Considering muscular fitness, evidence suggests it also plays a mediating role in the association between MVPA and inhibitory control (Bao et al., 2024). In summary, engagement in vigorous‐intensity physical activity may provide greater benefits to inhibitory control than lower intensity physical activity. Furthermore, cross‐sectional findings demonstrate that preadolescent children with greater engagement in MVPA in total exhibit a cognitive processing pattern distinct from their less active peers, which is also marked by differences in attentional resource allocation and conflict resolution during inhibitory control tasks involving stimulus conflict than less active peers (Ludyga et al., 2024).

Beyond the intensity of physical activity, accumulating evidence suggests that not all types of physical activity exert comparable effects on inhibitory control, with interventions enriched by cognitive demands yielding more pronounced improvements in this domain (Álvarez‐Bueno et al., 2017; Ludyga et al., 2020). This further suggests that categorizing sports by these characteristics can help predict their potential to enhance inhibitory control. Sport constitutes a specialized form of physical activity characterized by formalized rules, institutional organization and often a competitive component (Caspersen et al., 1985; Khan et al., 2012; Thivel et al., 2018). In this context, sports can be differentiated by the type of skills that are demanded in training and/or competition. Open‐skill sports, such as ball games and combat sports, require participants to respond within a rapidly changing, unpredictable and externally paced environment, necessitating continuous adaptation of their responses to external cues (Formenti et al., 2021). In contrast, closed‐skill sports, such as swimming or gymnastics, are defined by relatively stable, predictable and self‐paced environments, where motor actions are executed according to consistent and pre‐learned movement patterns (Formenti et al., 2021; Wang et al., 2013). The beneficial profile of open‐skill sports in fostering inhibitory control is assumed to extend to favourable patterns of classroom behaviour.

### Interindividual differences: the role of sex

While specific physical activity patterns and sport engagements have high potential to influence inhibitory control and on‐task behaviour, it remains unclear whether this potential is independent from interindividual differences. In this respect, sex differences may further modulate these associations, yet they have rarely been considered. Boys tend to exhibit physical activity patterns that may be more conducive to the development of inhibitory control. Specifically, they tend to have higher MVPA levels both in general (Peral‐Suárez et al., 2020; Renninger et al., 2021) and during school (Sasayama et al., 2025). In addition, the association between vigorous‐intensity physical activity and both cardiorespiratory and muscular fitness appears stronger in boys than in girls (Ávila‐García et al., 2020; Denton et al., 2013). Furthermore, boys show a greater preference for open‐skill sports in general (Peral‐Suárez et al., 2020). Moreover, while baseline sex differences in inhibitory control remain inconclusive (Gaillard et al., 2021), exercise interventions fostering inhibitory control appear to be more effective in male compared to female participants (Ludyga et al., 2020). However, girls outperform boys with regard to the time on task in the classroom (Godwin et al., 2016; Golaszewski et al., 2021).

We aimed to investigate the sex‐specific association of physical activity (including sports) and on‐task behaviour in preadolescent children, while considering intensity and type of sports engagement. We further examined a potential mediation of this association by inhibitory control. We hypothesized that open‐skill sports and vigorous‐intensity physical activity have a stronger positive association with inhibitory control compared to moderate‐intensity physical activity and closed‐skill sports or unspecific physical activities. Given that boys tend to engage more frequently in open‐skill sports and MVPA (Peral‐Suárez et al., 2020), and that previous exercise interventions targeting inhibitory control have shown greater effectiveness in boys (Ludyga et al., 2020), we hypothesized that the association between physical activity and both inhibitory control and on‐task behaviour would be stronger in boys than in girls.

## METHODS

### Study design and participants

This study presents a cross‐sectional analysis of baseline data from the NEXOS^2^ randomized controlled trial (German Clinical Trials Register, no. DRKS00033864), which investigated the acute neurophysiological effect of exercise with and without cooperative activities on social‐cognitive abilities in preadolescent school children. Participants were recruited through contact with school administrations in north‐western Switzerland, followed by the provision of informational events for legal guardians. Inclusion criteria comprised an age between >9 and ≤13 years and corrected‐to or normal vision. Participants were excluded if they were unable to engage in exercise; had an acute or chronic medical condition that could compromise safety during exercise; or were currently receiving pharmacological treatment, behavioural intervention or social skills training for a mental health disorder, as reported by their parents. Legal guardians and participants were fully informed about the study and written informed consent was obtained. The study protocol was approved by the local ethics committee (EKNZ, project ID: 2024‐00515) and complied with the Helsinki Declaration and its amendments.

### Procedure

Data assessment was performed class‐wise during the morning hours on a regular school day across 11 classes in 5 schools. Initially, classroom behaviour was recorded during a regular school lesson to assess on‐task behaviour. Participants then performed a computer‐based Flanker task. Subsequently, they filled in a 7‐day physical activity protocol, reported socio‐economic status (Family Affluence Scale: FAS) and psychopathology (Strength and Difficulties Questionnaire: SDQ). Finally, anthropometric data (body height and weight) were collected.

### Measures

#### Type and intensity of physical activity

MVPA was assessed using the 7‐Day Physical Activity Recall questionnaire since only protocols (in comparison to accelerometery) allow a classification into open‐ and closed‐skill sports (Nigg et al., 2020). The 7‐Day Physical Activity Recall questionnaire is a valid and reliable instrument in comparison to accelerometer measurements (Zuazagoitia et al., 2014), also in children (Sallis et al., 1992). Participants reported the type and the duration of MVPA for each day of the past regular school week. Moderate‐intensity physical activity was defined as activity that leads to noticeable shortness of breath, while vigorous‐intensity activity was defined as activity intense enough to induce sweating. The protocols were considered valid if participants were able to recall the entire 7‐day period and reported no injuries or other conditions that may have limited their participation in physical activity. Physical education classes were excluded from the analysis, as they were equal for all children and thus did not contribute to explaining any variance. In addition to the extraction of minutes spent in moderate‐intensity physical activity and vigorous‐intensity physical activity, we also assessed time spent in open‐ (e.g., playing soccer) and closed‐skill sports (e.g., running) and unspecific MVPA (e.g., participating in youth association) within the past week. The category ‘unspecific MVPA’ refers to activities reported in the 7‐Day Physical Activity Recall questionnaire that could not be clearly classified as either open‐ or closed‐skill sports. As the questionnaire captures only activities performed at moderate to vigorous intensity, all reported activities within this category were classified as MVPA. The categorization was performed by trained experts.

#### Inhibitory control

A computerized Flanker task adapted for children was administered with E‐Prime 3.0.3.214 (PST, Pittsburg, PA) to assess inhibitory control. Visual stimuli were five white fish presented against a black background. Participants were instructed to focus on the centrally presented target fish and to indicate its viewing direction as quickly and accurately as possible by pressing a corresponding button. The target fish was flanked by four additional fish, which were oriented either in the same direction as the target (congruent trials) or in the opposite direction (incongruent trials). The order of the congruent and incongruent trials was randomized. The session began with four example stimuli, followed by 12 practice trials. Participants then completed two blocks, each consisting of 36 trials. Following an inter‐stimulus interval of 900–1300 ms (random variation), visual stimuli were displayed until a response was collected or the presentation time reached 900 ms. Average reaction time (on response‐correct trials) and accuracy rates were calculated for each condition. Outcomes on incongruent trials reflected inhibitory control and were considered for statistical analyses.

#### On‐task behaviour

On‐task classroom behaviour during a regular school lesson was recorded using two video cameras positioned to capture participants' actions and eye gaze from two distinct angles. The children were not explicitly informed about the objective of the video recordings, which enhances the ecological validity. The same 6‐min segment of a regular school lesson was analysed for each child of this class, ensuring identical observation periods across participants. This segment was evaluated in 10‐s intervals, with each interval coded as either on‐task or off‐task behaviour. This resulted in 36 observations per child, conducted independently by two trained experimenters. If the discrepancies between the two raters were higher than 20%, a third experimenter was engaged. On‐task behaviour was defined as visual attention directed at the teacher or learning materials, as well as any verbal or motor actions that complied with classroom rules and were appropriate to the learning context. Deviating behaviour was evaluated as off‐task. The outcome was the mean number of on‐task observations. This video‐based assessment approach is consistent with methods used in prior research (Ludyga et al., 2022; Mahar et al., 2006).

### Statistical analysis

Statistical analyses were performed with SPSS Statistics 29.0.2.0 and Amos 28 Graphics (IBM Corp., Armonk, USA). Descriptive data (mean, standard deviation) were reported for age, body weight, body mass index (BMI), FAS, SDQ, both moderate and vigorous physical activity, open‐ and closed‐skill and unspecific MVPA, Flanker task performance during congruent and incongruent trials as well as on‐task behaviour. Prior to main analyses, the variable on‐task behaviour was z‐standardized within schools to account for differences in the implementation of the classroom observations. Subsequently, two different path models were calculated in boys, girls and all participants. Model 1 (Figure 1) investigated the association between moderate‐intensity and vigorous‐intensity physical activity and on‐task behaviour and its potential mediation by inhibitory control indicated by accuracy during incongruent trials of the Flanker task. Model 2 (Figure 2) examined the association between open‐ and closed‐skill sports as well as unspecific physical activities and on‐task behaviour and its potential mediation by inhibitory control. As age has been related to cognitive functioning (Leon‐Carrion et al., 2004; Ludyga et al., 2016; Macdonald et al., 2014) and to on‐task behaviour (Van Loon & Oeri, 2023) and BMI influences physical activity participation (Renninger et al., 2021), all models accounted for these variables. Similarly, reaction time on incongruent trials served as an additional covariate to control for potential speed‐accuracy trade‐offs. Both models for the total sample were controlled for sex. With regard to FAS and SDQ, zero‐order correlations showed no statistically significant relations of these variables with the outcomes of interest, so that both were not accounted for in the main analyses. Covariances between predictors and covariates were estimated. Missing data were treated using the maximum likelihood method. On‐task behaviour data were missing for 15.4% of the sample (*n* = 22 out of 142), while data for inhibitory control and physical activity were 99% complete. *T*‐tests were used to examine the hypothesis that a regression coefficient differed from zero, with statistical significance indicated by *p* ≤ .05. Model fit was assessed using the chi‐square statistic (Hu & Bentler, 1999), with acceptable levels at χ^2^/df <3.0 for the planned sample size (Iacobucci, 2010; Kline, 2016).

**FIGURE 1:**
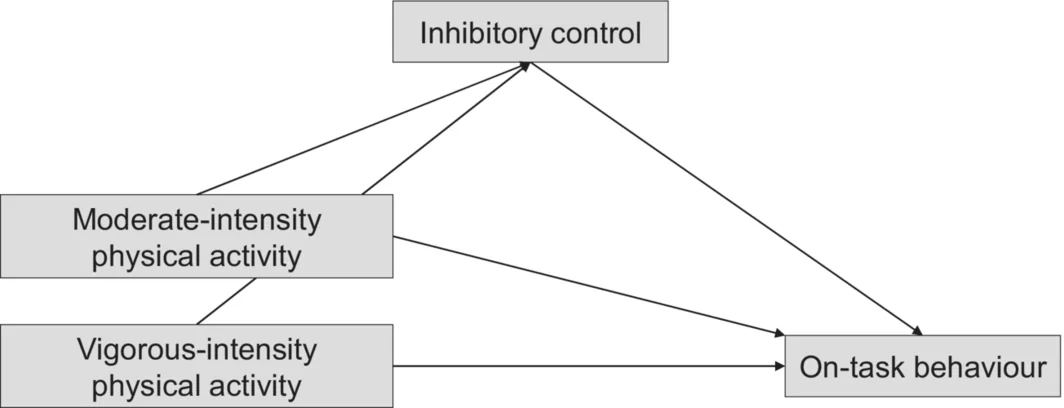
Model 1 investigating the association between moderate‐intensity and vigorous‐intensity physical activity and on‐task behaviour and its potential mediation by inhibitory control, controlled for age, body mass index and mean reaction time of the incongruent trials as well as sex in the total sample.

**FIGURE 2:**
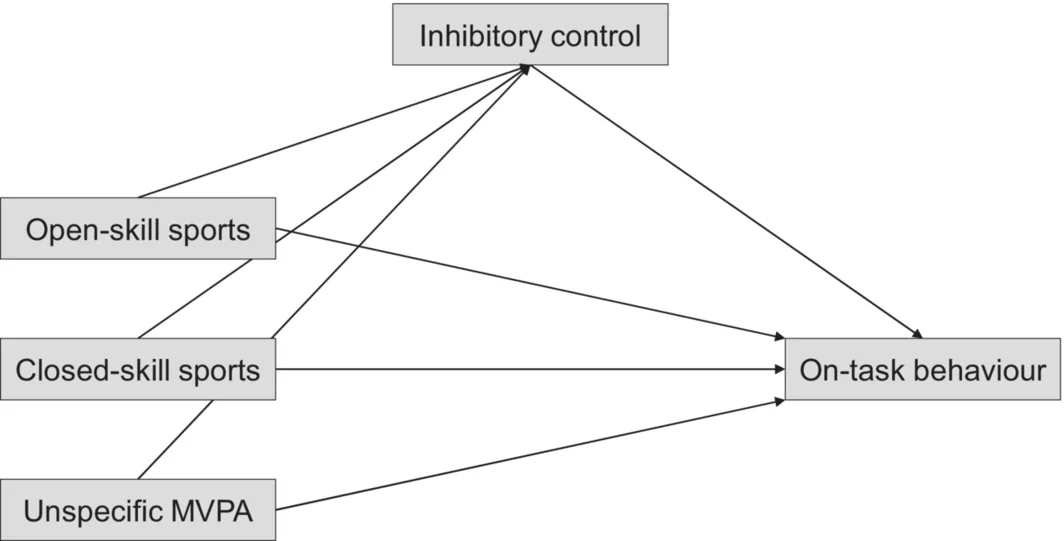
Model 2 investigating the association between open‐ and closed‐skill sports and unspecific moderate‐to‐vigorous physical activity (MVPA) and on‐task behaviour and its potential mediation by inhibitory control, controlled for age, body mass index and mean reaction time of the incongruent trials as well as sex in the total sample.

## RESULTS

Data from 142 children (47.2% boys and 52.8% girls) and five different schools were included in the analysis. Participants' characteristics, physical activity and sports participation, performance on the Flanker task and on‐task behaviour are shown in Table 1.

**TABLE 1:** Sex‐specific participant characteristics.

	Boys (*n* = 67)		Girls (*n* = 75)		All (*n* = 142)	
	M	SD	M	SD	M	SD
Age (years)	11.6	0.5	11.5	0.7	11.6	0.6
Body weight (kg)	45.2	12.3	45.5	11.0	45.4	11.6
Body mass index (kg/m^2^)	19.1	4.1	19.3	3.6	19.2	3.8
FAS (score)	6.9	1.6	7.0	1.7	6.9	1.7
SDQ (score)	13.1	5.4	12.3	5.1	12.7	5.3
MVPA (min/day)	66.4	54.0	38.5	26.9	51.7	44.1
MPA (min/day)	29.6	30.8	17.8	22.0	23.4	27.1
VPA (min/day)	36.8	44.1	20.7	23.0	28.3	35.5
Open‐skill sports (min/day)	34.9	36.1	11.4	18.9	22.5	30.6
Closed‐skill sports (min/day)	18.0	28.7	23.8	24.4	21.1	26.6
Unspecific MVPA (min/day)	9.5	22.2	4.1	8.5	6.7	16.6
Incongruent ACC (%)	88.2	11.4	90.9	11.7	89.6	11.6
Congruent ACC (%)	91.4	9.8	92.3	10.9	91.9	10.4
Incongruent RT (ms)	461.4	60.1	489.3	58.9	476.1	60.9
Congruent RT (ms)	446.4	60.0	474.9	58.0	461.4	60.5
On‐task behaviour (%)	76.5	17.2	80.4	17.0	78.5	17.1

Abbreviations: ACC, accuracy; FAS, Family Affluence Scale; MPA, moderate‐intensity physical activity; MVPA, moderate‐to‐vigorous physical activity; RT, reaction time SDQ; Strength and Difficulties Questionnaire; VPA, vigorous‐intensity physical activity.

### Intensity of physical activity

In Model 1 (Table 2), the covariance between moderate‐intensity physical activity and vigorous‐intensity physical activity was not statistically significant in the combined sample (*β* = −.03, *p* = .74) and among boys (*β* = .01, *p* = .94), but in girls (*β* = −.29, *p* < .05), indicating that higher levels of moderate‐intensity physical activity are associated with lower levels of vigorous‐intensity physical activity, or vice versa, in girls only. Path analysis revealed no associations between physical activity intensity and inhibitory control or on‐task behaviour or between inhibitory control and on‐task behaviour in the combined and sex‐specific analysis.

**TABLE 2:** Model 1 investigating the association between moderate‐intensity and vigorous‐intensity physical activity and on‐task behaviour and its potential mediation by inhibitory control, controlled for age and BMI as well as sex in the total sample.

Model 1	Boys (*n* = 67)		Girls (*n* = 75)		All (*n* = 142)	
	*β*	*p*	*β*	*p*	*β*	*p*
Incongruent ACC ← MPA	−.04	.71	−.01	.97	−.07	.42
Incongruent ACC ← VPA	.17	.16	.01	.94	.06	.44
Incongruent ACC ← Incongruent RT	−.24*	.047	−.30*	.009	−.23*	.005
On‐task behaviour ← Incongruent ACC	.06	.65	−.02	.84	.00	.97
On‐task behaviour ← MPA	.17	.19	−.05	.67	.07	.44
On‐task behaviour ← VPA	.6	.66	.18	.16	.12	.19
On‐task behaviour ← Age	−.18	.17	−.25*	.036	−.20*	.02
On‐task behaviour ← BMI	−.07	.58	−.11	.57	−.09	.32
On‐task behaviour ← Sex					.25*	.007

Abbreviations: ACC, accuracy; BMI, body mass index; MPA, moderate‐intensity physical activity; RT, reaction time; VPA, vigorous‐intensity physical activity.

* Significant *p*‐value.

### Sports type

In Model 2 (Table 3), referring to the total sample, higher levels of open‐skill sports were significantly associated with lower levels of closed‐skill sports (*β* = −.23, *p* < .01) and lower levels of unspecific MVPA (*β* = −.18, *p* < .05), whereas there was no significant association between closed‐skill sports and unspecific MVPA (*β* = .01, *p* = .89). Among boys, the covariance between open‐ and closed‐skill sports (*β* = −.15, *p* = .24) and between closed‐skill sports and unspecific MVPA (*β* = .02, *p* = .87) was not significantly related. In contrast, higher levels of open‐skill sports were associated with lower levels of unspecific MVPA (*β* = −.29, *p* < .05) in boys. In girls, greater engagement in open‐skill sports was associated with lower levels of closed‐skill sports (*β* = −.35, *p* < .01), whereas there was no significant relation between closed‐skill sports and unspecific MVPA (*β* = .07, *p* = .56) and between open‐skill sports and unspecific MVPA (*β* = −.05, *p* = .65).

**TABLE 3:** Model 2 investigating the association between open‐ and closed‐skill sports and unspecific MVPA and its potential mediation by inhibitory control, controlled for age and BMI as well as sex in the total sample.

Model 2	Boys (*n* = 67)		Girls (*n* = 75)		All (*n* = 142)	
	*β*	*p*	*β*	*p*	*β*	*p*
Incongruent ACC ← Open‐skill Sports	.07	.56	.04	.73	−.02	.81
Incongruent ACC ← Closed‐skill Sports	.11	.35	.05	.69	.08	.37
Incongruent ACC ← Unspecific MVPA	.04	.73	−.11	.33	−.05	.56
Incongruent ACC ← Incongruent RT	−.25*	.036	−.30*	.009	−.26*	.002
On‐task behaviour ← Incongruent ACC	.02	.85	−.02	.84	−.00	.99
On‐task behaviour ← Open‐skill Sports	.27*	.047	.09	.50	.18	.07
On‐task behaviour ← Closed‐skill Sports	.11	.41	.25*	.05	.16	.07
On‐task behaviour ← Unspecific MVPA	.01	.96	−.19	.12	−.05	.59
On‐task behaviour ← Age	−.15	.24	−.23	.06	−.18*	.032
On‐task behaviour ← BMI	−.11	.39	−.13	.28	−.12	.17
On‐task behaviour ← Sex					.25*	.009

Abbreviations: ACC, accuracy; BMI, body mass index; INC, incongruent trial; MVPA, moderate‐to‐vigorous physical activity; RT, reaction time.

* Significant *p*‐value.

Within the combined sample of boys and girls, time spent in the different sport types was not associated with inhibitory control, nor was inhibitory control related to on‐task behaviour. However, time spent in open‐ and closed‐skill sports was positively related to on‐task behaviour in the classroom with a tendency towards significance (*β* = .18, *p* = .07 and *β* = .16, *p* = .07 respectively), suggesting that greater engagement in these sport types was associated with higher levels of on‐task behaviour. Sex‐specific path analysis for boys indicated no association between sport types and inhibitory control, nor between inhibitory control and on‐task behaviour. However, the results showed a significant positive relationship between open‐skill sports and on‐task behaviour, indicating that higher levels of open‐skill sports are linked to greater on‐task behaviour in the classroom (*β* = .27, *p* < .05). In contrast, closed‐skill sports and unspecific MVPA did not appear to be related to on‐task behaviour. In girls, the path analysis revealed a statistically significant positive relationship between closed‐skill sports and on‐task behaviour, demonstrating that greater time spent in closed‐skill sports is associated with higher levels of on‐task behaviour (*β* = .25, *p* = .05). No relation was found between open‐skill sports or unspecific MVPA and on‐task behaviour. Time spent in different sport types was not associated with inhibitory control and inhibitory control showed no relationship with on‐task behaviour.

A list of the activities mentioned in the 7‐Day Physical Activity Recall questionnaire and their classification into open‐skill and closed‐skill sports can be found in Table S1 of the supplementary material. Within the sex‐specific models, model fit is acceptable for both Model 1 (χ^2^/df = 21.25/15 = 1.42) and for Model 2 (χ^2^/df = 21.53/15 = 1.44). Similarly, acceptable fit indices were observed in the models for the total sample (Model 1: χ^2^/df = 20.31/9 = 2.26 and Model 2: χ^2^/df = 19.54/9 = 2.17).

## DISCUSSION

Path analyses restricted to either girls or boys did not support an association between the intensity of physical activity and on‐task behaviour. However, significant sex‐specific associations were identified between the type of sports engagement and on‐task behaviour. Among boys, greater engagement in open‐skill sports was related to higher levels of on‐task behaviour, whereas in girls, higher participation in closed‐skill sports was linked to greater on‐task behaviour. In both models, type of sports and intensity of physical activity were not associated with inhibitory control, arguing against a mediating role of this component of executive function.

Concerning the relationship between physical activity and inhibitory control, our results add to the inconclusive evidence, as neither the intensity nor the type of physical activity was associated with inhibitory control. A similar lack of associations between MVPA and inhibitory control has also been reported in previous studies. For example, Pindus et al. (2019) conducted a cross‐sectional investigation of the relationship between MVPA measured via accelerometry, with a particular focus on vigorous‐intensity physical activity, and inhibitory control in 8–10‐year‐old children. Their findings revealed no significant associations between daily MVPA or in particular between vigorous‐intensity physical activity and inhibitory control. Domazet et al. (2016) also found no association between MVPA, measured with accelerometry, and inhibitory control in school children aged 12–14 years. Due to the cross‐sectional study design, dynamic changes in both MVPA and inhibitory control might be missed. For example, if a child has only recently experienced a change in physical activity levels, whether an increase or decrease, effects on inhibitory control may not be immediately evident and may emerge only after a temporal delay. With regard to the lack of an association between sport type and inhibitory control in our study, it is important to note that Formenti et al. (2021) allocated children to an open‐skill sports, closed‐skill sports or to a sedentary group, based on the sports the children mainly engaged in. Similarly, Wang et al. (2013) investigated inhibitory control in tennis players and swimmers, with athletes engaging in high training volumes and non‐exercisers. However, such strict categorization does not reflect the typical activity patterns of children, who often participate in multiple sport types (Agans & Geldhof, 2012). Therefore, we adopted an approach that allowed different sport types, with path analyses employed to assess the relative contributions of open and closed‐skill sports to on‐task behaviour.

Using this multi‐sport types, path‐analytic approach, we identified sex‐specific relations of sport type and on‐task behaviour, providing an indication that specific sport types have unique predictive value for on‐task behaviour in boys and girls. These sex‐specific patterns warrant consideration of the mechanisms that may account for these associations. Our results could imply that both sexes use different strategies to allocate attentional resources to remain on task. In this respect, meta‐analytical findings demonstrate that open‐skill sports are related to increased cognitive flexibility in children, whereas engagement in both open‐ and closed‐skill sports is equally related to better working memory performance (Lai et al., 2024). Based on this, boys may rely more on cognitive flexibility, which is the ability to adapt thinking and behaviour in response to changing demands. In the classroom, this might help children to shift their attention between different tasks and points of interest without losing focus. Girls engaging in closed‐skill sports may use working memory to a greater extent. Even when faced with a temporal distraction, they can rely on working memory to re‐engage with the task by integrating new demands with previously learned information, thereby facilitating a more efficient return to task‐focused behaviour. Besides that, descriptive data showed that boys engaged predominantly in open‐skill sports compared to their total MVPA, whereas girls spent most of their MVPA time in closed‐skill sports. Therefore, it is likely that the distribution pattern corresponds to sex‐related preferences in sport participation. From a hedonistic perspective on physical activity (Ekkekakis & Zenko, 2016), it may be posited that children engage in self‐chosen leisure activities that bring them pleasure, which could influence their affective state in a positive way (Hyde et al., 2011; Li et al., 2022). Engaging in enjoyable physical activities may contribute to the replenishment or preservation of self‐control resources that are taxed during the school day (Baumeister et al., 2007). In turn, greater availability of these resources may be associated with more favourable on‐task behaviour (Audiffren & André, 2015; Muraven & Baumeister, 2000). The lack of mediation by inhibitory control does not contradict this assumption, as inhibitory control is only one component of self‐control (Miyake & Friedman, 2012).

In addition to sport type, we examined the association between physical activity intensity and on‐task behaviour. In contrast to previous studies showing an association of MVPA and on‐task behaviour (Brusseau & Burns, 2018; Carlson et al., 2015; Ludyga et al., 2022; Mahar et al., 2006), our findings do not support that the overall dose spent in either moderate‐intensity physical activity or vigorous‐intensity physical activity explains variance in time on task in the classroom. As MVPA is simply the product of different physical activities, this discrepancy might be due to varying contents of the overall MVPA across studies as stereotypical activities that count towards MVPA depend on the context and the culture (Bann et al., 2019; Hulteen et al., 2017). For example, while Brusseau and Burns (2018) studied children attending low‐income schools in a capital city in the southwestern United States, our study focused on children living in a rural region in Switzerland. Contextual differences may result in variations in MVPA behaviour (type, intensity as well as number and duration of bouts), which are not adequately captured when MVPA is treated as a single, undifferentiated measure. While this may explain the deviation of our results from data collected in other countries, it may not explain the discrepancy from our previous study that showed a positive association between MVPA and on‐task behaviour in children from the same region (Ludyga et al., 2022). However, a notable difference between these studies is the age range (9–13 vs. 10–16 years). During adolescence, most children have typically entered or even completed puberty, and they experienced a school transition to different educational levels. These developmental and contextual changes may influence the relationship between MVPA and on‐task behaviour. For instance, pubertal onset, especially relative to peers, is associated with emotional, social and academic difficulties (Duncan et al., 1985; Vijayakumar et al., 2024; Warren & Yu, 2015). It is accompanied by shifts in social roles and peer relationships, which may contribute to these difficulties. As peers reach comparable levels of physical development, such effects tend to diminish (Warren & Yu, 2015). Early maturing boys display more deviant behaviour, while early maturing girls exhibit greater dissatisfaction with their body image and show more depressive symptoms (Duncan et al., 1985; Vijayakumar et al., 2024). In this regard, emotional competencies might play a key role in helping children adapt to developmental changes at this age and may therefore be more relevant to on‐task behaviour than executive function.

The absence of a mediating role for inhibitory control in the relationship between MVPA and on‐task behaviour, in contrast to the findings of Ludyga et al. (2022), strengthens the assumption that mechanisms beyond inhibitory control should be considered in this age group. Self‐regulation is described as the ability ‘to monitor and modulate cognition, emotion and behaviour, to accomplish one's goal and/or to adapt to the cognitive and social demands of specific situations’ (Berger et al., 2007, p. 257). Future research might consider investigating emotion regulation as a mediating variable. It refers to the capacity to manage emotional responses in ways that support goal‐directed behaviour, including the ability to suppress, amplify, sustain or adjust emotional arousal (Eisenberg & Spinrad, 2004; Schlesier et al., 2019). The classroom is an emotionally charged environment, where students face challenges such as receiving criticism, being evaluated, making mistakes, maintaining concentration and engaging with difficult tasks. Emotional responses may also be triggered by classroom disruptions, adherence to rules and situations perceived as boring, exciting or disappointing (Kurki et al., 2015). The appropriate use of emotion regulation strategies influences learning behaviour (Kurki et al., 2015). Better emotion regulation is linked not only to academic success, engagement, motivation and problem management (Schlesier et al., 2019) but also to positive teacher–student relationships and to improved attention and more effective processing of new information presented by the teacher (Graziano et al., 2007). Existing evidence supports the notion that habitual physical exercise enhances emotion regulation abilities. Exercise appears to foster emotional flexibility which is defined as the ability to recover effectively from emotional challenges (Bernstein et al., 2019, 2020).

### Strengths, limitations and future directions

The strength of this study is the high ecological validity due to the objective, video‐based assessment of on‐task behaviour, in which all children were evaluated for the same time period by two, respectively, three independent examiners, and the capture of real‐life classroom dynamics, as on‐task behaviour was assessed during a regular school lesson with their real teacher. Nevertheless, the following limitations should be considered when interpreting the results. First, the associations were derived from cross‐sectional data; therefore, no causal inferences are allowed. In this context, it is also important to consider the possibility of a reverse causal relationship, whereby higher abilities of staying on‐task may promote greater participation in sports. Second, the 7‐Day Physical Activity Recall questionnaire depends on the children's ability to recall physical activity of the past week and to evaluate the experienced intensity. Furthermore, self‐report measures are inherently susceptible to bias, including social desirability bias (Nigg et al., 2020). However, only the protocol allowed the identification of types of physical activity. Accelerometers do not capture all forms of children's physical activity. For instance, they tend to underestimate activities such as cycling and those involving primarily upper‐body movement. Additionally, they must be removed for water‐based or contact sports. Third, the classification of sport types into open‐ and closed‐skill categories is well established, it may attenuate meaningful variability (e.g., coordinative demands) within categories. For instance, coordinatively demanding closed‐skill activities (e.g., hip hop dancing) are grouped together with less coordinatively complex activities such as cycling or running, despite evidence suggesting that higher coordinative demands are more strongly associated with cognitive benefits (Ludyga et al., 2020). Fourth, not all elements of sport participation are necessarily beneficial for cognitive functioning. Activities involving head impacts (e.g., headers in football or strikes in combat sports) may be neutral or even detrimental to cognitive outcomes (Herold et al., 2026). As such exposures are characteristic of several open‐skill sports included in this study, the present classification does not differentiate between potentially beneficial and adverse components. This may have introduced additional variability in the observed associations and should be considered when interpreting the findings. Fifth, by using the Flanker task, we were able to measure inhibitory control, but separable components of executive functions, such as cognitive flexibility and working memory, were not assessed. Thus, we cannot rule out if sports type influences on‐task behaviour indirectly by specific cognitive abilities. Sixth, on‐task behaviour might have been affected by context‐specific factors that we did not account for, including the student‐teacher relationship, the classroom environment, or the general interest in the subject. Seventh, we did not account for individual differences in chronotype or sleepiness, which may also influence on‐task behaviour. Prior research suggests that both chronotype and daily levels of sleepiness are associated with attentional functioning in children and adolescents (Escribano & Díaz‐Morales, 2014). Future research should further aim to develop a more in‐depth understanding of activity contexts (Agans & Geldhof, 2012), as coaching style, team culture, competitive vs. recreational settings and team vs. individual sports, as well as the dose–response characteristics between physical activity and on‐task behaviour. In this context, the use of ambulatory assessment methods may be valuable, as they can reduce recall bias and provide more fine‐grained information on physical activity behaviour (Reichert et al., 2020). However, it should also be acknowledged that such approaches are resource‐intensive and may be challenging to implement in child populations (Meter & Ehrenreich, 2022). In addition, future studies would benefit from the application of micro‐longitudinal designs, which allow for the examination of short‐term within‐person dynamics and may help to better capture temporal associations between physical activity, cognitive functions and on‐task behaviour (Herold et al., 2026).

## CONCLUSION

The association between physical activity and classroom behaviour is influenced by the type rather than its intensity. Specifically, open‐skills sports are related to more frequent on‐task behaviour in boys, whereas participation in closed‐skill sports is linked with favourable classroom behaviour in girls. While it remains unclear whether these sex‐specific relations are due to differing physiological adaptation profiles or sports preferences, they provide an indication that interventions targeting classroom behaviour should be tailored to boys'‐ and girls'‐specific needs.

## AUTHOR CONTRIBUTIONS

**Melanie Berger:** Project administration; writing – original draft; formal analysis. **Toru Ishihara:** Conceptualization; writing – review and editing. **Keita Kamijo:** Conceptualization; writing – review and editing. **Markus Gerber:** Resources; writing – review and editing. **Sebastian Ludyga:** Funding acquisition; conceptualization; project administration; formal analysis; writing – review and editing.

## CONFLICT OF INTEREST STATEMENT

There are no conflicts of interest to declare.

## Supporting information


Data S1

## Data Availability

The data that support the findings of this study are available from the corresponding author upon reasonable request.

## References

[bjep70123-bib-0001] Agans, J. P. , & Geldhof, G. J. (2012). Trajectories of participation in athletics and positive youth development: The influence of sport type. Applied Developmental Science, 16(3), 151–165. 10.1080/10888691.2012.697792

[bjep70123-bib-0002] Álvarez‐Bueno, C. , Pesce, C. , Cavero‐Redondo, I. , Sánchez‐López, M. , Martínez‐Hortelano, J. A. , & Martínez‐Vizcaíno, V. (2017). The effect of physical activity interventions on Children's cognition and metacognition: A systematic review and meta‐analysis. Journal of the American Academy of Child & Adolescent Psychiatry, 56(9), 729–738. 10.1016/j.jaac.2017.06.012 28838577

[bjep70123-bib-0003] Audiffren, M. , & André, N. (2015). The strength model of self‐control revisited: Linking acute and chronic effects of exercise on executive functions. Journal of Sport and Health Science, 4(1), 30–46. 10.1016/j.jshs.2014.09.002

[bjep70123-bib-0004] Ávila‐García, M. , Baena‐Ogalla, N. , Huertas‐Delgado, F. J. , Tercedor, P. , & Villa‐González, E. (2020). The relationship between physical activity levels, cardiorespiratory fitness and academic achievement school‐age children from southern Spain. Sustainability, 12(8), 3459. 10.3390/su12083459

[bjep70123-bib-0005] Bann, D. , Scholes, S. , Fluharty, M. , & Shure, N. (2019). Adolescents' physical activity: Cross‐national comparisons of levels, distributions and disparities across 52 countries. International Journal of Behavioral Nutrition and Physical Activity, 16(1), 141. 10.1186/s12966-019-0897-z PMC6937925 31888652

[bjep70123-bib-0006] Bao, R. , Leahy, A. A. , Lubans, D. R. , Diallo, T. M. O. , Beauchamp, M. R. , Smith, J. J. , Hillman, C. H. , & Wade, L. (2024). Mediators of the association between physical activity and executive functions in primary school children. Journal of Sports Sciences, 42(21), 2029–2038. 10.1080/02640414.2024.2422203 39467694

[bjep70123-bib-0007] Baumeister, R. F. , Vohs, K. D. , & Tice, D. M. (2007). The strength model of self‐control. Current Directions in Psychological Science, 16(6), 351–355.

[bjep70123-bib-0008] Berger, A. , Kofman, O. , Livneh, U. , & Henik, A. (2007). Multidisciplinary perspectives on attention and the development of self‐regulation. Progress in Neurobiology, 82(5), 256–286. 10.1016/j.pneurobio.2007.06.004 17651888

[bjep70123-bib-0009] Bernstein, E. E. , Curtiss, J. E. , Wu, G. W. Y. , Barreira, P. J. , & McNally, R. J. (2019). Exercise and emotion dynamics: An experience sampling study. Emotion, 19(4), 637–644. 10.1037/emo0000462 29999384

[bjep70123-bib-0010] Bernstein, E. E. , Heeren, A. , & McNally, R. J. (2020). A network approach to understanding the emotion regulation benefits of aerobic exercise. Cognitive Therapy and Research, 44(1), 52–60. 10.1007/s10608-019-10039-6

[bjep70123-bib-0011] Brusseau, T. A. , & Burns, R. D. (2018). Physical activity, health‐related fitness, and classroom behavior in children: A discriminant function analysis. Research Quarterly for Exercise and Sport, 89(4), 411–417. 10.1080/02701367.2018.1519521 30285569

[bjep70123-bib-0012] Carlson, J. A. , Engelberg, J. K. , Cain, K. L. , Conway, T. L. , Mignano, A. M. , Bonilla, E. A. , Geremia, C. , & Sallis, J. F. (2015). Implementing classroom physical activity breaks: Associations with student physical activity and classroom behavior. Preventive Medicine, 81, 67–72. 10.1016/j.ypmed.2015.08.006 26297105

[bjep70123-bib-0013] Caspersen, C. J. , Powell, K. E. , & Christenson, G. M. (1985). Physical activity, exercise, and physical fitness: Definitions and distinctions for health‐related research. Public Health Reports, 100(2), 126–131. PMC1424733 3920711

[bjep70123-bib-0014] Denton, S. J. , Trenell, M. I. , Plötz, T. , Savory, L. A. , Bailey, D. P. , & Kerr, C. J. (2013). Cardiorespiratory fitness is associated with hard and light intensity physical activity but not time spent sedentary in 10–14 year old schoolchildren: The HAPPY study. PLoS One, 8(4), e61073. 10.1371/journal.pone.0061073 PMC3618292 23577192

[bjep70123-bib-0015] Domazet, S. L. , Tarp, J. , Huang, T. , Gejl, A. K. , Andersen, L. B. , Froberg, K. , & Bugge, A. (2016). Associations of physical activity, sports participation and active commuting on mathematic performance and inhibitory control in adolescents. PLoS One, 11(1), e0146319. 10.1371/journal.pone.0146319 PMC4699746 26727211

[bjep70123-bib-0016] Duncan, P. D. , Ritter, P. L. , Dornbusch, S. M. , Gross, R. T. , & Merrill Carlsmith, J. (1985). The effects of pubertal timing on body image, school behavior, and deviance. Journal of Youth and Adolescence, 14(3), 227–235. 10.1007/BF02090320 24301178

[bjep70123-bib-0017] Eisenberg, N. , & Spinrad, T. L. (2004). Emotion‐related regulation: Sharpening the definition. Child Development, 75(2), 334–339. 10.1111/j.1467-8624.2004.00674.x 15056187

[bjep70123-bib-0018] Ekkekakis, P. , & Zenko, Z. (2016). Escape from cognitivism: Exercise as hedonic experience. In Sport and exercise psychology research (pp. 389–414). Elsevier. 10.1016/B978-0-12-803634-1.00018-2

[bjep70123-bib-0019] Escribano, C. , & Díaz‐Morales, J. F. (2014). Daily fluctuations in attention at school considering starting time and chronotype: An exploratory study. Chronobiology International, 31(6), 761–769. 10.3109/07420528.2014.898649 24679224

[bjep70123-bib-0020] Formenti, D. , Trecroci, A. , Duca, M. , Cavaggioni, L. , D'Angelo, F. , Passi, A. , Longo, S. , & Alberti, G. (2021). Differences in inhibitory control and motor fitness in children practicing open and closed skill sports. Scientific Reports, 11(1), 4033. 10.1038/s41598-021-82698-z PMC7889632 33597630

[bjep70123-bib-0021] Gaillard, A. , Fehring, D. J. , & Rossell, S. L. (2021). A systematic review and meta‐analysis of behavioural sex differences in executive control. European Journal of Neuroscience, 53(2), 519–542. 10.1111/ejn.14946 32844505

[bjep70123-bib-0022] García‐Hermoso, A. , Ezzatvar, Y. , Ramírez‐Vélez, R. , Olloquequi, J. , & Izquierdo, M. (2021). Is device‐measured vigorous physical activity associated with health‐related outcomes in children and adolescents? A systematic review and meta‐analysis. Journal of Sport and Health Science, 10(3), 296–307. 10.1016/j.jshs.2020.12.001 PMC8167335 33285309

[bjep70123-bib-0023] Godwin, K. E. , Almeda, M. V. , Seltman, H. , Kai, S. , Skerbetz, M. D. , Baker, R. S. , & Fisher, A. V. (2016). Off‐task behavior in elementary school children. Learning and Instruction, 44, 128–143. 10.1016/j.learninstruc.2016.04.003

[bjep70123-bib-0024] Golaszewski, N. , Bartholomew, J. , Errisuriz, V. , Korinek, E. , & Jowers, E. (2021). Predictors of on‐task behaviors: Evaluating student‐level characteristics. Health Behavior and Policy Review, 8(2), 159–167. 10.14485/hbpr.8.2.6 PMC8562880 34734099

[bjep70123-bib-0025] Graziano, P. A. , Reavis, R. D. , Keane, S. P. , & Calkins, S. D. (2007). The role of emotion regulation in children's early academic success. Journal of School Psychology, 45(1), 3–19. 10.1016/j.jsp.2006.09.002 PMC3004175 21179384

[bjep70123-bib-0026] Herold, F. , Pontifex, M. B. , Gronwald, T. , & Heath, M. (2026). Does a header a day keep the positive effects of physical activity on cognitive performance away? A call to consider physical activity‐related head impacts as a moderator variable. Sport Sciences for Health, 22(1), 4. 10.1007/s11332-025-01627-z

[bjep70123-bib-0027] Hu, L. , & Bentler, P. M. (1999). Cutoff criteria for fit indexes in covariance structure analysis: Conventional criteria versus new alternatives. Structural Equation Modeling: A Multidisciplinary Journal, 6(1), 1–55. 10.1080/10705519909540118

[bjep70123-bib-0028] Hulteen, R. M. , Smith, J. J. , Morgan, P. J. , Barnett, L. M. , Hallal, P. C. , Colyvas, K. , & Lubans, D. R. (2017). Global participation in sport and leisure‐time physical activities: A systematic review and meta‐analysis. Preventive Medicine, 95, 14–25. 10.1016/j.ypmed.2016.11.027 27939265

[bjep70123-bib-0029] Hyde, A. L. , Conroy, D. E. , Pincus, A. L. , & Ram, N. (2011). Unpacking the feel‐good effect of free‐time physical activity: Between‐ and within‐person associations with pleasant–activated feeling states. Journal of Sport and Exercise Psychology, 33(6), 884–902. 10.1123/jsep.33.6.884 PMC4144026 22262710

[bjep70123-bib-0030] Iacobucci, D. (2010). Structural equations modeling: Fit indices, sample size, and advanced topics. Journal of Consumer Psychology, 20(1), 90–98. 10.1016/j.jcps.2009.09.003

[bjep70123-bib-0031] Kao, S.‐C. , Drollette, E. S. , Scudder, M. R. , Raine, L. B. , Westfall, D. R. , Pontifex, M. B. , & Hillman, C. H. (2017). Aerobic fitness is associated with cognitive control strategy in preadolescent children. Journal of Motor Behavior, 49(2), 150–162. 10.1080/00222895.2016.1161594 PMC6125153 27715503

[bjep70123-bib-0032] Khan, K. M. , Thompson, A. M. , Blair, S. N. , Sallis, J. F. , Powell, K. E. , Bull, F. C. , & Bauman, A. E. (2012). Sport and exercise as contributors to the health of nations. The Lancet, 380(9836), 59–64. 10.1016/S0140-6736(12)60865-4 22770457

[bjep70123-bib-0033] Kline, R. B. (2016). Principles and practice of structural equation modeling (4th ed.). The Guilford Press.

[bjep70123-bib-0034] Kurki, K. , Järvelä, S. , Mykkänen, A. , & Määttä, E. (2015). Investigating children's emotion regulation in socio‐emotionally challenging classroom situations. Early Child Development and Care, 185(8), 1238–1254. 10.1080/03004430.2014.988710

[bjep70123-bib-0035] Lai, J. , Zhang, Z. , Ni, G. , & Luo, Y. (2024). The influence of open‐skill and closed‐skill sports on executive functions: A systematic review. British Journal of Hospital Medicine, 85(7), 1–16. 10.12968/hmed.2024.0168 39078904

[bjep70123-bib-0036] Leon‐Carrion, J. , García‐Orza, J. , & Pérez‐Santamaría, F. J. (2004). Development of the inhibitory component of the executive functions in children and adolescents. International Journal of Neuroscience, 114(10), 1291–1311. 10.1080/00207450490476066 15370187

[bjep70123-bib-0037] Li, J. , Huang, Z. , Si, W. , & Shao, T. (2022). The effects of physical activity on positive emotions in children and adolescents: A systematic review and meta‐analysis. International Journal of Environmental Research and Public Health, 19(21), 14185. 10.3390/ijerph192114185 PMC9657539 36361067

[bjep70123-bib-0038] Ligeza, T. S. , Raine, L. B. , Pontifex, M. B. , Wyczesany, M. , Kramer, A. F. , & Hillman, C. H. (2024). Cognitive benefits of higher cardiorespiratory fitness in preadolescent children are associated with increased connectivity within the cingulo‐opercular network. Scientific Reports, 14(1), 21193. 10.1038/s41598-024-72074-y PMC11390878 39261550

[bjep70123-bib-0039] Ludyga, S. , Gerber, M. , Brand, S. , Holsboer‐Trachsler, E. , & Pühse, U. (2016). Acute effects of moderate aerobic exercise on specific aspects of executive function in different age and fitness groups: A meta‐analysis. Psychophysiology, 53(11), 1611–1626. 10.1111/psyp.12736 27556572

[bjep70123-bib-0040] Ludyga, S. , Gerber, M. , Brand, S. , Möhring, W. , & Pühse, U. (2022). Do different cognitive domains mediate the association between moderate‐to‐vigorous physical activity and adolescents' off‐task behaviour in the classroom? British Journal of Educational Psychology, 92(1), e12445. 10.1111/bjep.12445 PMC9291588 34254665

[bjep70123-bib-0041] Ludyga, S. , Gerber, M. , Herold, F. , Schwarz, A. , Looser, V. N. , & Hanke, M. (2024). Cortical hemodynamics and inhibitory processing in preadolescent children with low and high physical activity. International Journal of Clinical and Health Psychology, 24(1), 100438. 10.1016/j.ijchp.2024.100438 PMC10788801 38226004

[bjep70123-bib-0042] Ludyga, S. , Gerber, M. , Pühse, U. , Looser, V. N. , & Kamijo, K. (2020). Systematic review and meta‐analysis investigating moderators of long‐term effects of exercise on cognition in healthy individuals. Nature Human Behaviour, 4(6), 603–612. 10.1038/s41562-020-0851-8 32231280

[bjep70123-bib-0043] Macdonald, J. A. , Beauchamp, M. H. , Crigan, J. A. , & Anderson, P. J. (2014). Age‐related differences in inhibitory control in the early school years. Child Neuropsychology, 20(5), 509–526. 10.1080/09297049.2013.822060 23909718

[bjep70123-bib-0044] Mahar, M. T. , Murphy, S. K. , Rowe, D. A. , Golden, J. , Shields, A. T. , & Raedeke, T. D. (2006). Effects of a classroom‐based program on physical activity and on‐task behavior. Medicine & Science in Sports & Exercise, 38(12), 2086–2094. 10.1249/01.mss.0000235359.16685.a3 17146314

[bjep70123-bib-0045] Martínez‐Gómez, D. , Welk, G. J. , Puertollano, M. A. , del‐Campo, J. , Moya, J. M. , Marcos, A. , Veiga, O. L. , & AFINOS Study Group . (2011). Associations of physical activity with muscular fitness in adolescents. Scandinavian Journal of Medicine & Science in Sports, 21(2), 310–317. 10.1111/j.1600-0838.2009.01036.x 20030783

[bjep70123-bib-0046] Meter, D. J. , & Ehrenreich, S. E. (2022). Child development in real time: The power of ambulatory assessment for investigating dynamic developmental processes and behavior longitudinally. In Advances in child development and behavior (Vol. 62, pp. 269–294). Elsevier. 10.1016/bs.acdb.2021.12.004 35249684

[bjep70123-bib-0047] Miyake, A. , & Friedman, N. P. (2012). The nature and Organization of Individual Differences in executive functions: Four general conclusions. Current Directions in Psychological Science, 21(1), 8–14. 10.1177/0963721411429458 PMC3388901 22773897

[bjep70123-bib-0048] Moffett, L. , & Morrison, F. J. (2020). Off‐task behavior in kindergarten: Relations to executive function and academic achievement. Journal of Educational Psychology, 112(5), 938–955. 10.1037/edu0000397

[bjep70123-bib-0049] Muraven, M. , & Baumeister, R. F. (2000). Self‐regulation and depletion of limited resources: Does self‐control resemble a muscle? Psychological Bulletin, 126(2), 247–259. 10.1037/0033-2909.126.2.247 10748642

[bjep70123-bib-0050] Nigg, C. R. , Fuchs, R. , Gerber, M. , Jekauc, D. , Koch, T. , Krell‐Roesch, J. , Lippke, S. , Mnich, C. , Novak, B. , Ju, Q. , Sattler, M. C. , Schmidt, S. C. E. , Van Poppel, M. , Reimers, A. K. , Wagner, P. , Woods, C. , & Woll, A. (2020). Assessing physical activity through questionnaires – A consensus of best practices and future directions. Psychology of Sport and Exercise, 50, 101715. 10.1016/j.psychsport.2020.101715 PMC7430559 32831643

[bjep70123-bib-0051] Peral‐Suárez, Á. , Cuadrado‐Soto, E. , Perea, J. M. , Navia, B. , López‐Sobaler, A. M. , & Ortega, R. M. (2020). Physical activity practice and sports preferences in a group of Spanish schoolchildren depending on sex and parental care: A gender perspective. BMC Pediatrics, 20(1), 337. 10.1186/s12887-020-02229-z PMC7339494 32635918

[bjep70123-bib-0052] Pindus, D. M. , Drollette, E. S. , Raine, L. B. , Kao, S.‐C. , Khan, N. , Westfall, D. R. , Hamill, M. , Shorin, R. , Calobrisi, E. , John, D. , Kramer, A. F. , & Hillman, C. H. (2019). Moving fast, thinking fast: The relations of physical activity levels and bouts to neuroelectric indices of inhibitory control in preadolescents. Journal of Sport and Health Science, 8(4), 301–314. 10.1016/j.jshs.2019.02.003 PMC6620425 31333883

[bjep70123-bib-0053] Pontifex, M. B. , Raine, L. B. , Johnson, C. R. , Chaddock, L. , Voss, M. W. , Cohen, N. J. , Kramer, A. F. , & Hillman, C. H. (2011). Cardiorespiratory fitness and the flexible modulation of cognitive control in preadolescent children. Journal of Cognitive Neuroscience, 23(6), 1332–1345. 10.1162/jocn.2010.21528 20521857

[bjep70123-bib-0054] Reichert, M. , Giurgiu, M. , Koch, E. D. , Wieland, L. M. , Lautenbach, S. , Neubauer, A. B. , Von Haaren‐Mack, B. , Schilling, R. , Timm, I. , Notthoff, N. , Marzi, I. , Hill, H. , Brüßler, S. , Eckert, T. , Fiedler, J. , Burchartz, A. , Anedda, B. , Wunsch, K. , Gerber, M. , … Liao, Y. (2020). Ambulatory assessment for physical activity research: State of the science, best practices and future directions. Psychology of Sport and Exercise, 50, 101742. 10.1016/j.psychsport.2020.101742 PMC7430559 32831643

[bjep70123-bib-0055] Renninger, D. , Sturm, D. J. , Marques, A. , Peralta, M. , Popovic, S. , Gardasevic, J. , Masanovic, B. , & Demetriou, Y. (2021). Physical activity and body‐mass‐index: Do family, friends and teachers restrain the risk for physical inactivity in adolescents? Sustainability, 13(13), 6992. 10.3390/su13136992

[bjep70123-bib-0056] Sallis, J. F. , Buono, M. J. , Roby, J. J. , Micale, F. G. , & Nelson, J. A. (1992). Seven‐day recall and other physical activity self‐reports in children and adolescents. Medicine and Science in Sports and Exercise, 25, 99–108. 10.1249/00005768-199301000-00014 8423762

[bjep70123-bib-0057] Sasayama, K. , Yasunebe, J. , & Adachi, M. (2025). Longitudinal changes and tracking of in‐school physical activity in primary school children: Four‐year longitudinal study. BMC Public Health, 25(1), 1041. 10.1186/s12889-025-22256-8 PMC11917022 40102823

[bjep70123-bib-0058] Schlesier, J. , Roden, I. , & Moschner, B. (2019). Emotion regulation in primary school children: A systematic review. Children and Youth Services Review, 100, 239–257. 10.1016/j.childyouth.2019.02.044

[bjep70123-bib-0059] Smith, J. J. , Eather, N. , Weaver, R. G. , Riley, N. , Beets, M. W. , & Lubans, D. R. (2019). Behavioral correlates of muscular fitness in children and adolescents: A systematic review. Sports Medicine, 49(6), 887–904. 10.1007/s40279-019-01089-7 30864143

[bjep70123-bib-0060] Spengler, M. , Damian, R. I. , & Roberts, B. W. (2018). How you behave in school predicts life success above and beyond family background, broad traits, and cognitive ability. Journal of Personality and Social Psychology, 114(4), 620–636. 10.1037/pspp0000185 29504796

[bjep70123-bib-0061] Thivel, D. , Tremblay, A. , Genin, P. M. , Panahi, S. , Rivière, D. , & Duclos, M. (2018). Physical activity, inactivity, and sedentary behaviors: Definitions and implications in occupational health. Frontiers in Public Health, 6, 288. 10.3389/fpubh.2018.00288 PMC6182813 30345266

[bjep70123-bib-0062] Van Loon, M. H. , & Oeri, N. S. (2023). Examining on‐task regulation in school children: Interrelations between monitoring, regulation, and task performance. Journal of Educational Psychology, 115(3), 446–459. 10.1037/edu0000781

[bjep70123-bib-0063] Vijayakumar, N. , Youssef, G. , Bereznicki, H. , Dehestani, N. , Silk, T. J. , & Whittle, S. (2024). The social determinants of emotional and behavioral problems in adolescents experiencing early puberty. Journal of Adolescent Health, 74(4), 674–681. 10.1016/j.jadohealth.2023.06.025 37665306

[bjep70123-bib-0064] Wang, C.‐H. , Chang, C.‐C. , Liang, Y.‐M. , Shih, C.‐M. , Chiu, W.‐S. , Tseng, P. , Hung, D. L. , Tzeng, O. J. L. , Muggleton, N. G. , & Juan, C.‐H. (2013). Open vs. closed skill sports and the modulation of inhibitory control. PLoS One, 8(2), e55773. 10.1371/journal.pone.0055773 PMC3572130 23418458

[bjep70123-bib-0065] Warren, D. , & Yu, M. (2015). Pubertal status and emotional, school and social functioning. LSAC Annual Statistical Report.

[bjep70123-bib-0066] Zuazagoitia, A. , Montoya, I. , Grandes, G. , Arietaleanizbeascoa, M. S. , Arce, V. , Martinez, V. , Sanchez, M. , & Sanchez, A. (2014). Reliability and validity of the 7‐day physical activity recall interview in a Spanish population. European Journal of Sport Science, 14(S1), S361–S368. 10.1080/17461391.2012.705332 24444230

