# Supplementary material for: Type and intensity of physical activity and their sex‐specific relation with classroom behaviour in preadolescent children
Source: Br J Educ Psychol. 2026 Aug 24;96(4):1846–61. doi: 10.1111/bjep.70123 (PMC13752627; doi:10.1111/bjep.70123)
Supplement: Supplementary file 1 [file BJEP-96-1846-s001.docx]

# **Supplementary material**

**Table 4**: Activities listed in the 7-Day Physical Activity Recall questionnaire, categorized as open-skill or closed-skill activities.

| **Open-skill sport** | **Closed-skill sport** |
| --- | --- |
| Aikido (Formenti et al., 2021) | Apparatus gymnastics (Heilmann et al., 2022) |
| Badminton (Formenti et al., 2021) | Ballet (Han & Jang, 2025) |
| Basketball (Formenti et al., 2021) | Curling |
| Boxing (Formenti et al., 2021) | Cycling and Unicycling (Formenti et al., 2021) |
| Football (Soccer) (Formenti et al., 2021) | Floor gymnastics (Heilmann et al., 2022) |
| Handball (Formenti et al., 2021) | Hiking (Formenti et al., 2021) |
| Judo (Formenti et al., 2021) | Hip-hop dancing |
| Karate (Formenti et al., 2021) | Horse riding |
| Kickboxing (Formenti et al., 2021) | Ice skating (Heilmann et al., 2022) |
| Roller Hockey (Formenti et al., 2021) | Running (Formenti et al., 2021) |
| Table Tennis (Formenti et al., 2021) | Shooting sports (Heilmann et al., 2022) |
| Taekwondo (Formenti et al., 2021) | Skiing |
| Tennis (Formenti et al., 2021) | Strength training (Heilmann et al., 2022) |
| Volleyball (Formenti et al., 2021) | Swimming (Formenti et al., 2021) |
|  | Track and field (Formenti et al., 2021) |
|  | Trampoline jumping |
|  | Yoga (Han & Jang, 2025) |
